# To accurately predict lymph node metastasis in patients with mass-forming intrahepatic cholangiocarcinoma by using CT radiomics features of tumor habitat subregions

**DOI:** 10.1186/s40644-025-00842-8

**Published:** 2025-02-26

**Authors:** Pengyu Chen, Zhenwei Yang, Peigang Ning, Hao Yuan, Zuochao Qi, Qingshan Li, Bo Meng, Xianzhou Zhang, Haibo Yu

**Affiliations:** 1 Department of Hepatobiliary Surgery, Henan University People’S Hospital, Henan Provincial People’S Hospital, Zhengzhou, China; 2 Department of Radiology, People’s Hospital of Zhengzhou University, Zhengzhou, China; 3 Department of Hepatobiliary Surgery, Henan Provincial People’s Hospital, Zhengzhou, China; 4 Department of Hepatobiliary Surgery, Henan Cancer Hospital, Zhengzhou, China

**Keywords:** Intrahepatic cholangiocarcinoma, Radiomics, Lymph node metastasis, Tumor habitat subregions, Machine learning

## Abstract

**Background:**

This study aims to introduce the concept of habitat subregions and construct an accurate prediction model by analyzing refined medical images, to predict lymph node metastasis (LNM) in patients with intrahepatic cholangiocarcinoma (ICC) before surgery, and to provide personalized support for clinical decision-making.

**Methods:**

Clinical, radiological, and pathological data from ICC patients were retrospectively collected. Using information from the arterial and venous phases of multisequence CT images, tumor habitat subregions were delineated through the K-means clustering algorithm. Radiomic features were extracted and screened, and prediction models based on different subregions were constructed and compared with traditional intratumoral models. Finally, a lymph node metastasis prediction model was established by integrating the features of several subregional models, and its performance was evaluated.

**Results:**

A total of 164 ICC patients were included in this study, 103 of whom underwent lymph node dissection. The patients were divided into LNM- and LNM + groups on the basis of lymph node status, and significant differences in white blood cell indicators were found between the two groups. Survival analysis revealed that patients with positive lymph nodes had significantly worse prognoses. Through cluster analysis, the optimal number of habitat subregions was determined to be 5, and prediction models based on different subregions were constructed. A comparison of the performance of each model revealed that the Habitat1 and Habitat5 models had excellent performance. The optimal model obtained by fusing the features of the Habitat1 and Habitat5 models had AUC values of 0.923 and 0.913 in the training set and validation set, respectively, demonstrating good predictive ability. Calibration curves and decision curve analysis further validated the superiority and clinical application value of the model.

**Conclusions:**

This study successfully constructed an accurate prediction model based on habitat subregions that can effectively predict the lymph node metastasis of ICC patients preoperatively. This model is expected to provide personalized decision support to clinicians and help to optimize treatment plans and improve patient outcomes.

**Supplementary Information:**

The online version contains supplementary material available at 10.1186/s40644-025-00842-8.

## Background

Intrahepatic cholangiocarcinoma (ICC), the second most common primary malignant tumor of the liver, accounts for approximately 15%−20% of primary liver cancers. Recently, its incidence has increased yearly, garnering increasing attention from the medical community [1, 2]. However, the invasiveness, recurrence and metastasis of ICC often lead to a poor prognosis [3]. Numerous studies have indicated that lymph node metastasis (LNM) is a critical factor affecting the long-term prognosis of ICC patients after surgery [4, 5]. Lymph node dissection is recommended as an important part of the surgical treatment of intrahepatic cholangiocarcinoma [6, 7]. This not only aids in determining the disease stage but also facilitates the development of more precise and personalized treatment strategies. Furthermore, with advances in medical technology, the application of neoadjuvant therapy and conversion therapy in ICC patients has gradually increased [8–10]. There may be a correlation between lymph node metastasis status and the efficacy of these treatments. Therefore, accurate preoperative assessment of lymph node metastasis status is crucial for improving patient survival rates. However, in previous clinical practice, we reported that many patients with intrahepatic cholangiocarcinoma do not undergo lymph node dissection, undoubtedly limiting doctors’ comprehensive understanding and precise treatment of the patient’s condition.

Tumor subregions refer to local areas within a tumor that exhibit distinct tissue structures and functional characteristics [11]. The formation of these subregions often stems from tumor heterogeneity, differences in vascular distribution, diversity in metabolic states, and complexity in gene expression patterns during tumor growth [12, 13]. In the field of medical imaging, for example, in the interpretation of computed tomography (CT) images, significant differences in density, morphology, or texture can be observed among tumor subregions [14, 15]. These differences are not merely imaging artifacts but reflect the biological characteristics of the tumor, such as necrotic areas, hemorrhagic foci, calcifications, and regions with varying cell proliferation activities. Lymph nodes, an important part of the human immune system, play crucial roles in the development and metastasis of tumors [16]. Therefore, exploring the relationships between imaging features of tumor subregions and lymph node metastasis is highly important for improving the accuracy of tumor diagnosis, optimizing treatment strategies, and enhancing prognostic evaluation.

Radiomics, with its profound ability to analyze medical imaging data, successfully extracts complex features that are difficult to capture with the naked eye and utilizes these features to construct precise models [17, 18]. Previous studies exploring the prediction of tumor lymph node metastasis have shown that the application of radiomics can significantly increase diagnostic accuracy [19]. Through precise analysis, doctors can gain a more comprehensive understanding of tumor growth and lymph node metastasis, thereby enhancing their knowledge of the patient’s condition.

Therefore, we have innovatively introduced the concept of using tumor microenvironment subregions to conduct a deep and refined analysis of medical imaging data. On this basis, we constructed a precise model for predicting lymph node metastasis in ICC patients before surgery. This model aims to guide clinicians in comprehensively evaluating the risk of lymph node metastasis in patients in the preoperative stage and provides more personalized clinical decision support.

## Methods

### Patients

We retrospectively collected detailed laboratory, imaging, and pathological data from ICC patients who were treated at Henan Provincial People’s Hospital and Henan Cancer Hospital from January 2018 to June 2023. Preoperative laboratory tests included routine blood tests, liver function tests, renal function tests, coagulation function tests, and hepatic virology tests to ensure a comprehensive assessment of the patients’ physical condition. Imaging data focused on enhanced CT provide crucial information for subsequent radiomic analysis.

The patients included in this study met the following criteria. First, they were diagnosed with intrahepatic cholangiocarcinoma confirmed by postoperative histopathological examination. Second, patients underwent enhanced CT examination, and the imaging data, including critical information such as arterial and venous phases, were clear and complete. Finally, for patients who underwent lymph node dissection, the diagnosis of their lymph nodes was based on histopathological examination results.

However, the following were exclusion criteria: poor-quality imaging data, which may affect the accuracy of radiomic analysis; concurrent diagnosis of other types of malignancies to prevent interference with the assessment of lymph node metastasis in intrahepatic cholangiocarcinoma; preoperative antitumor treatment, which may alter the imaging appearance of the tumor; and severe comorbidities that may affect surgical procedures and postoperative recovery.

After rigorous screening, a total of 164 patients were included in this study. Among them, 103 patients underwent intraoperative lymph node dissection, whereas 61 patients did not. The lymph node dissection rate was 62.8%. In the lymph node dissection group, there were 51 LNM- patients and 52 LNM + patients. We divided the dataset into training and testing sets at a 6:4 ratio and employed random cross-validation to construct the prediction model, aiming to increase the stability and accuracy of the model.

Overall survival (OS) was calculated as the interval between the date of surgery and the date of death or the last follow-up. Follow-up was conducted through various methods, including phone calls, outpatient visits, and hospital re-examinations, until July 2022. The OS data for 99 patients were obtained through follow-up, including 67 patients in the lymph node dissection group and 32 patients in the nondissection group.

**Fig. 1:**
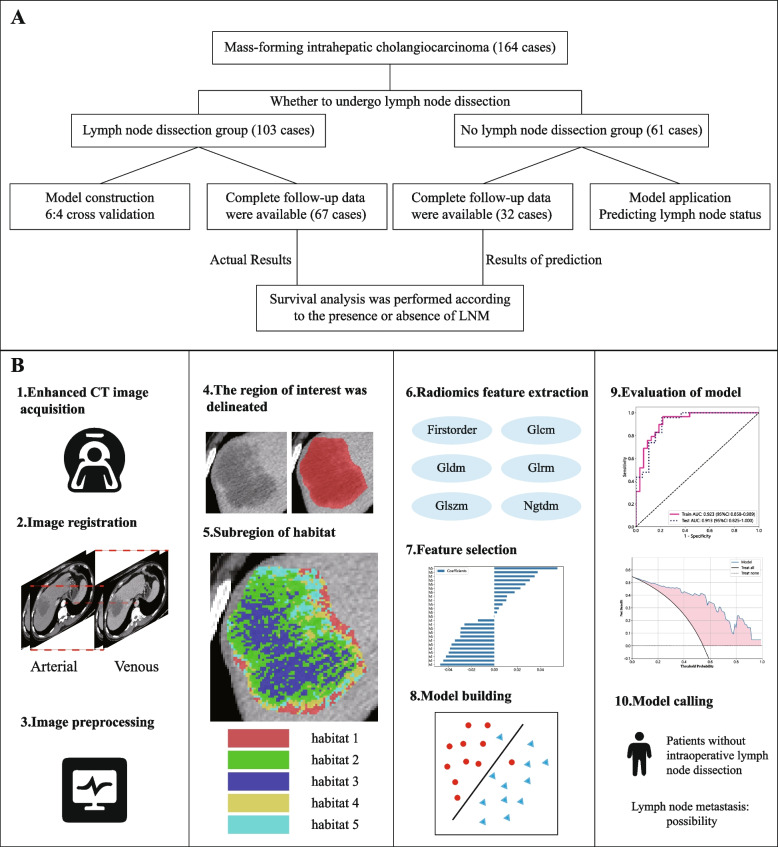
**A** Work flow chart. **B** Model construction flow chart

This study strictly adhered to the principles of the Declaration of Helsinki and was approved by the Ethics Committees of Henan University People’s Hospital and Henan Cancer Hospital (approval numbers Ref No. 2023–012 and Ref No. 2023–203). The entire study process is illustrated in Fig. 1.

### Image preprocessing

In multisequence CT images, the arterial and venous phases were selected, with an image slice thickness of 1 mm or 1.25 mm. More scan details are provided in Supplementary material 1. To eliminate the influence of patient differences on image quality, window width and window level standardization were applied to the CT images, setting a window width and window level suitable for abdominal observation (50/350 HU). This also ensured that the target tissues could be displayed clearly and accurately. Voxels in CT images can vary in size due to differences in scanning conditions or patient body types. However, voxels of different sizes are not conducive to model training and can affect the accuracy of subsequent image processing and analysis. By resampling the images to 1*1*1mm^3^, the voxel sizes were normalized to the same size, making subsequent analysis more accurate and reliable. To spatially align two images of the same subject acquired at different times for comparison, we used the “registration moving image layer” function in ITK-SNAP software. With the venous phase image as a reference, we performed a combination of automatic and manual registration on the arterial phase image. By precisely adjusting parameters such as the spatial position, rotation, and scaling of the image layers, we found the best match between the two images to ensure that they were spatially aligned.

Finally, we imported the processed images into ITK-SNAP software and invited two experienced radiologists to use the brush tool for layer-by-layer fine delineation, accurately defining the regions of interest (ROIs). Reader 1 (clinical experience: 13 years) labeled the tumor, and Reader 2 (clinical experience: 22 years) reviewed the delineation boundaries. Furthermore, mass-forming intrahepatic cholangiocarcinoma with well-defined boundaries was intentionally chosen to minimize the margin of error in delineating tumor boundaries. This step provided a solid data foundation for subsequent analysis, ensuring the accuracy and reliability of the study.

### Delineation of habitat subregions

On the basis of the grayscale information in the arterial and venous phases in the CT images, we used a K-means clustering algorithm to segment the tumor region into different parts. The goal of clustering was to identify different subregions within the extent of the tumor region [20, 21]. First, the gray level information of the tumor region was extracted from the CT image and converted into a gray value matrix. In such a matrix, each small square, which we call a voxel, corresponds to a unique gray value. Next, these grayscale values are clustered. To determine the optimal number of clusters (K value), we adopted the Calinski–Harabasz index (CH index) as the evaluation criterion. This index considers both the closeness within clusters and the separation between clusters to help find the most appropriate value of K. Using the selected best K value and the feature matrix, the center of the cluster is updated by iteration, and each voxel is assigned to the nearest cluster. We subsequently analyzed the clustering results of the K-means algorithm to ensure that each cluster was reasonable and distinguishable within the gray value space. Finally, we used visualization tools to superpose the clustering results onto the original CT images. In this way, we were able to visualize the distributions and properties of the different subregions more intuitively. Additional information on habitat subregional division is provided in Supplementary material 2.

### Feature extraction

Following the Image Biomarker Standardization Initiative, this process uses the PyRadiomics package to efficiently extract many imaging features that describe tumor characteristics from medical images. Since venous phase images have higher image quality, the contrast between tumor tissue and normal tissue is greater. Therefore, feature extraction in this study was based on venous sequences. The extracted features include first-order gradient features, shape features, and texture features. To enable a more comprehensive understanding of image content and attributes and to provide strong support for subsequent analysis and decision-making, we also included features extracted after various image filtering transformations, such as Gaussian‒Laplacian transformation, wavelet transformation, and square root filtering.

### Feature selection and model construction

To eliminate the scale differences between high-dimensional radiomic features, we used the z score method to normalize the features before performing feature selection. For features with high repetitiveness, we calculated the correlation between features using Spearman’s rank correlation coefficient. When the correlation coefficient between a feature and other features was greater than 0.9, only that feature was retained. To maximize the descriptive ability of the features, a greedy recursive deletion strategy was adopted for feature selection, which involved deleting the feature with the highest redundancy in the current set each time. After that, we applied LASSO to further reduce the dimensionality of the features. On the basis of the regularization weight λ, LASSO shrank some regression coefficients to zero, effectively eliminating irrelevant features and retaining those with nonzero coefficients in the regression model. Finally, these features were combined into a radiomic signature, and a radiomic score was calculated for each patient from a linear combination of the retained features.

### Statistics and methods

Statistical analysis was performed using SPSS 26.0 and Python 3.11 software. The chi-square test was used to analyze the risk factors for lymph node metastasis. Factors with significant differences in univariate analysis were included in multivariate analysis, for which binary logistic regression models were used. Kaplan‒Meier curves were used for survival analysis, and the log-rank test was used to compare the differences between the two groups of Kaplan‒Meier curves to test whether the differences in survival rates between the groups were significant. LASSO analysis reduced the complexity of the model and achieved feature selection by introducing L1 regularization. The classification performance of the model was evaluated by plotting ROC curves and calculating area under the curve (AUC) values. Decision curve analysis (DCA) was used to evaluate the clinical utility of the prediction model. Calibration plots were used to assess the agreement between the predicted values from the prediction model and the observed values.

## Results

### Patient characteristics and outcomes

In this study, 103 patients with primary ICC who underwent surgical lymph node dissection were divided into a node-negative group and a node-positive group according to the degree of lymph node metastasis. Seventy-four of the patients (71.8%) were HBsAg negative. Twenty-nine patients (28.2%) had abnormal alpha-fetoprotein (AFP) levels. Seventy-seven of the patients (74.8%) had abnormal carbohydrate antigen 199 (CA199) levels. After a detailed comparative analysis of the preoperative laboratory test results, we found a significant difference in the white blood cell count between the node-negative and node-positive groups (Table 1). The clinical data of the 61 patients who did not undergo lymph node dissection are shown in Supplementary Table 1.

**Table 1:** The clinical data of lymph node metastasis negative group and lymph node metastasis positive group were compared

Variable		LNM (-)	LNM ( +)	Chi-square	*P*-value
HBsAg	-	36(48.6)	38(51.4)	0.004	0.95
	+	15(51.7)	14(48.3)		
AFP	< 7 ng/ml	39(52.7)	35(47.3)	0.664	0.41
	> = 7 ng/ml	12(41.4)	17(58.6)		
CEA	< 5 ng/ml	36(55.4)	29(44.6)	1.834	0.18
	> = 5 ng/ml	15(39.5)	23(60.5)		
CA199	< 27 ng/ml	17(65.4)	9(34.6)	2.706	0.10
	> = 27 ng/ml	34(44.2)	43(55.8)		
albumin	< 43.2 g/l	28(42.4)	38(57.6)	2.948	0.09
	> = 43.2 g/l	23(62.2)	14(37.8)		
GGT	< 97.9 u/l	30(55.6)	24(44.4)	1.188	0.28
	> = 97.9 u/l	21(42.9)	28(57.1)		
ALP	< 208.2 u/l	46(54.1)	39(45.9)	3.136	0.08
	> = 208.2 u/l	5(27.8)	13(72.2)		
INR	< 1.025	30(44.1)	38(55.9)	1.740	0.19
	> = 1.025	21(60.0)	14(40.0)		
APTT	< 29.1 s	28(56.0)	22(44.0)	1.170	0.28
	> = 29.1 s	23(43.4)	30(56.6)		
WBC	< 6.7 10*9/l	17(37.0)	29(63.0)	4.376	0.04
	> = 6.7 10*9/l	34(59.6)	23(40.3)		
NC	< 4.2 10*9/l	21(42.9)	28(57.1)	1.188	0.28
	> = 4.2 10*9/l	30(55.6)	24(44.4)		
LC	< 1.8 10*9/l	34(44.2)	43(55.8)	2.706	0.10
	> = 1.8 10*9/l	17(65.4)	9(34.6)		
PLT	< 280.5 10*9/l	44(54.3)	37(45.7)	2.662	0.10
	> = 280.5 10*9/l	7(31.8)	15(68.2)		
creatinine	< 51.5 umol/l	8(33.3)	16(66.6)	2.488	0.11
	> = 51.5 umol/l	43(54.4)	36(45.6)		

*HBsAg* Hepatitis B Surface Antigen, *AFP* Alpha-Fetoprotein, *CEA* Carcinoembryonic Antigen, *CA199* Carbohydrate Antigen 199, *GGT* Gamma-Glutamyl Transferase, *ALP* Alkaline Phosphatase, *INR* International Normalized Ratio, *APTT* Activated Partial Thromboplastin Time, *WBC* White blood cell count, *NC* Neutrophil count, *LC* Lymphocyte count, *PLT* Platelet count

**Fig. 2:**
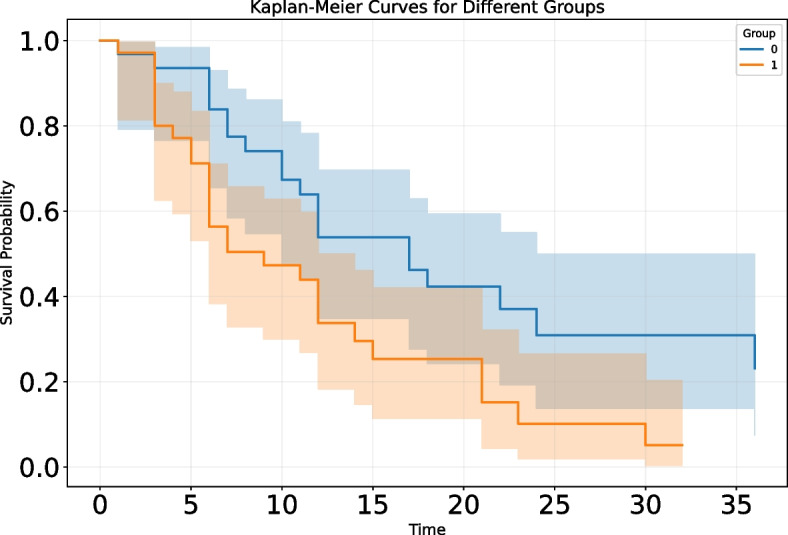
Association between lymph node status and overall survival in patients with intrahepatic cholangiocarcinoma. Group 0: negative for lymph node metastasis; Group 1: positive for lymph node metastasis

To explore the relationship between lymph node status and survival rates in ICC patients, we used the Kaplan‒Meier survival analysis method to evaluate the overall survival rates of the two groups and drew survival curves accordingly. Through statistical verification using the log-rank test, we observed a significant correlation between lymph node status and survival time in ICC patients, with a statistically significant *p* value of 0.01. Specifically, ICC patients with positive lymph nodes had significantly worse prognoses than those with negative lymph nodes. This was visually reflected in the survival curves, which showed a clear trend of separation (Fig. 2). Further analysis of the median survival time revealed that the median survival time for patients in the LNM-negative group was 17 ± 2.99 months, whereas that for patients in the LNM-positive group was 9 ± 3.45 months, and the overall median survival time was 12 ± 0.94 months.

### Optimal partitioning of habitat regions

**Fig. 3:**
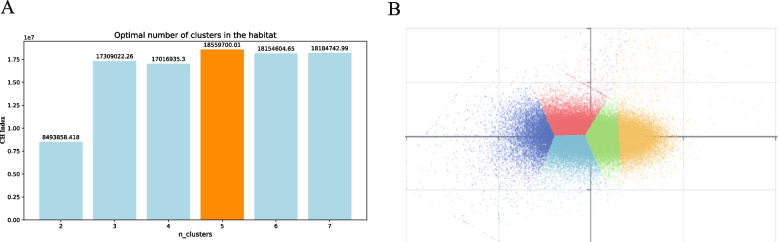
Optimal division of habitat areas. **A** The optimal number of habitats was determined on the basis of the maximum value of the CH index. **B** Visualization of the clustering results

To accurately partition habitat subregions and explore their predictive value for lymph node status in ICC patients, a clustering analysis method was employed to determine the optimal number of subregions. In the clustering process, a total of 30,222,209 voxels were extracted from the arterial phase and venous phase CT images, and different clustering schemes with different numbers of subregions were tested. Comparative analysis showed that the CH index reached its maximum value when the number of clusters was 5 (Fig. 3A). This suggests that dividing the habitat into 5 subregions can best preserve the intrinsic structure and information of the data while reducing noise and redundancy. To visualize the clustering results more intuitively, the sample_ratio parameter was used for downsampling visualization, retaining 1% of the original data for display (Fig. 3B). Visualization clearly revealed that these voxels could be effectively divided into 5 distinct subregions, each with unique characteristics and distribution patterns. These subregions not only reflect the heterogeneity within the tumor but also provide an important foundation for subsequent feature extraction and model building.

### Comparison of intratumor model performance with models based on habitat subregions

**Fig. 4:**
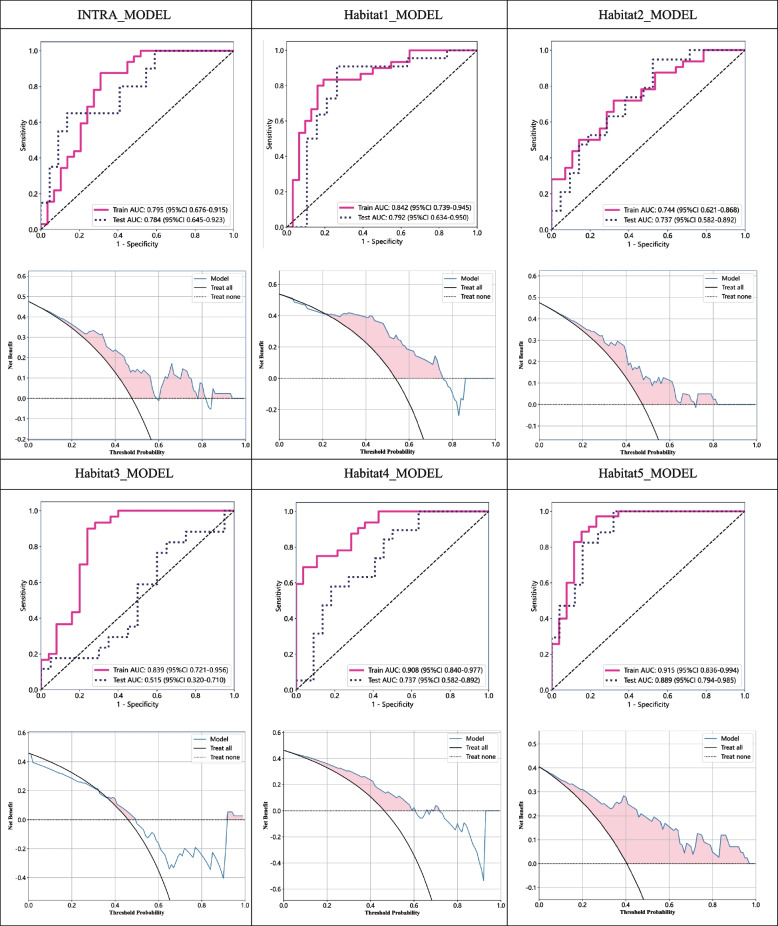
Comparison of the intratumoral models with the five habitat subregional models. The top graph shows the receiver operating characteristic curve (ROC) of the corresponding model, and the bottom graph shows the decision curve analysis (DCA)

To achieve accurate prediction of lymph node status in ICC patients, we designed and implemented multiple predictive models. These models encompassed an intratumor model based on the entire tumor region (INTRA-MODEL) and models based on different habitat subregions (Habitat1, Habitat2, Habitat3, Habitat4, Habitat5-MODEL). During model construction, we fully considered the heterogeneity within the tumor and attempted to extract features from different subregions to improve the prediction accuracy. To evaluate the performance of the models, we employed a rigorous cross-validation method, ensuring that the models exhibited stable predictive capabilities across different datasets to further increase their generalizability. The experimental results (Table 2) demonstrated that the models based on habitat subregions presented significant advantages in terms of prediction accuracy. Among them, the H1 and H5 models had good performance in terms of the AUC value and accuracy in both the training and test sets. To visualize the predictive performance of different models more intuitively, we also plotted corresponding predictive performance comparison charts (Fig. 4). When the Delong test was applied to compare the prediction performance of the INTRA-MODEL model with that of different habitat subregion models, we obtained detailed results, as detailed in Supplementary Table 2. Notably, the difference in prediction performance between the INTRA-MODEL and the H5 model was statistically significant (*P* = 0.011), indicating a clear difference in prediction ability between the two models. However, the results of the Delong test revealed no statistically significant difference between the INTRA-MODEL and H1 model (*P* = 0.598). Although the H1 model itself showed good prediction performance, its advantage over the INTRA-MODEL was not obvious, and the prediction accuracies of the two models tended to be similar.

**Table 2:** Comparison of intra-tumor model performance with models based on habitat subregions

Model-name	Group	AUC (95% CI)	Accuracy	Sensitivity	Specificity	PPV	NPV
**Intra-model**	train	0.795(0.676–0.915)	0.770	0.844	0.690	0.750	0.800
	test	0.784(0.645–0.923)	0.738	0.600	0.864	0.800	0.704
**Habitat1-model**	train	0.842(0.739–0.945)	0.803	0.800	0.806	0.800	0.806
	test	0.792(0.634–0.950)	0.805	0.864	0.737	0.792	0.824
**Habitat2-model**	train	0.744(0.621–0.868)	0.683	0.687	0.679	0.710	0.655
	test	0.737(0.582–0.892)	0.675	0.895	0.476	0.607	0.833
**Habitat3-model**	train	0.839(0.721–0.956)	0.818	0.867	0.760	0.812	0.826
	test	0.515(0.320–0.710)	0.541	0.765	0.350	0.500	0.636
**Habitat4-model**	train	0.908(0.840–0.977)	0.800	0.656	0.964	0.955	0.711
	test	0.737(0.582–0.892)	0.683	0.526	0.818	0.714	0.667
**Habitat5-model**	train	0.915(0.836–0.994)	0.869	0.943	0.769	0.846	0.909
	test	0.889(0.794–0.985)	0.786	0.941	0.680	0.667	0.944

*AUC* Area Under the Curve, *PPV* Positive Predictive Value, *NPV* Negative Predictive Value

### Optimal model and its clinical application value

**Fig. 5:**
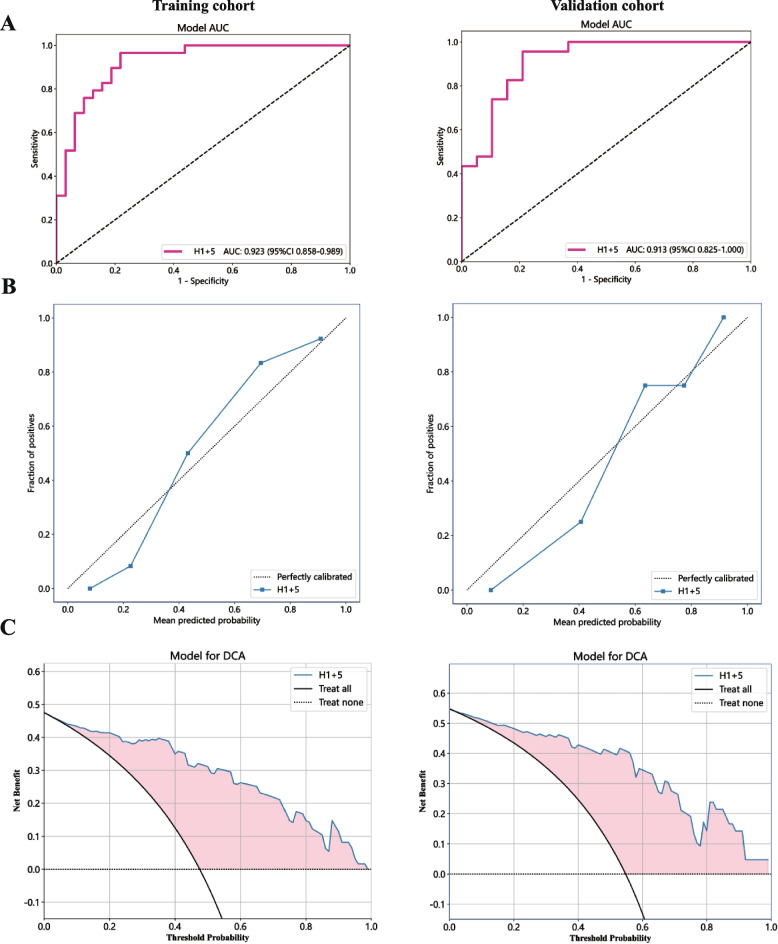
Performance evaluation of the final model. **A** Receiver operating characteristic (ROC) curve. **B** Calibration curve. **C**Decision curve

After comparative analysis, we integrated the features of Habitat1 and Habitat5, aiming to further improve the prediction accuracy. Through a rigorous feature selection process, we identified 25 features, including 10 from Habitat1 and 15 from Habitat5. To evaluate the model performance, we divided the patient data into a 6:4 ratio for model training and validation. By conducting 100 cross-validations, we obtained a stable model with excellent performance. The ROC curves of the model on the training and validation sets are shown in Fig. 5A, with AUC values of 0.923 (95% CI 0.858–0.989) and 0.913 (95% CI 0.825–1.000), respectively, indicating the model’s good predictive ability. Additionally, we analyzed the model’s calibration curve (Fig. 5B) and decision curve (Fig. 5C). The calibration curve fit well, suggesting a high degree of consistency between the model’s predictions and the actual results. The results of the Hosmer–Lemeshow test in the training group and the test group were 0.852 and 0.398, respectively. The decision curve demonstrated significant positive benefits during the prediction process, further validating the model’s superiority.

**Fig. 6:**
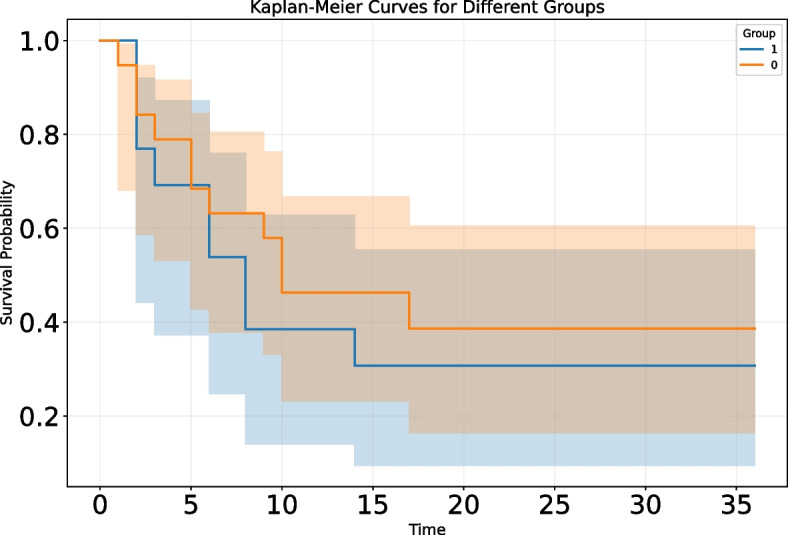
Association between predicted lymph node status and overall survival in patients with intrahepatic cholangiocarcinoma (patients without lymph node dissection). Group0: negative for lymph node metastasis; Group1: positive for lymph node metastasis

After the model was constructed, we applied it to clinical scenarios for ICC patients who did not undergo lymph node dissection during surgery. Through the model, we successfully predicted the lymph node status of these patients. To ensure the reliability of the model predictions, we conducted in-depth validation using survival follow-up data from some patients. Specifically, we divided the patients into two groups: those predicted to have positive metastasis and those predicted to have negative metastasis. A detailed survival analysis was performed. The results revealed that the median survival time for patients in the LNM- group was 10 ± 4.83 months, whereas it was 8 ± 2.19 months for patients in the positive group, and the overall median survival time was 9 ± 2.204 months. As illustrated by the Kaplan‒Meier curve in Fig. 6, we observed some differences in survival time between the two groups.

## Discussion

This study conducted an in-depth exploration of the lymph node status in patients with mass-forming intrahepatic cholangiocarcinoma. We not only analyzed the relationship between lymph node status and patient survival from a clinical perspective but also constructed an innovative prediction model using optimal habitat region segmentation to achieve accurate prediction of lymph node status in ICC patients.

Through group comparisons of ICC patients who underwent intraoperative lymph node dissection, we found a significant difference in overall survival between the LNM- group and the LNM + group. This finding not only further confirms the important role of lymph node status in the prognostic evaluation of ICC patients but also reveals the increased risk of death faced by patients with positive lymph nodes, which is consistent with previous findings in the medical field [22, 23].

The significant difference in white blood cell counts between the LNM- and LNM + groups is noteworthy. White blood cells, as important immune cells in the human body, can reflect the immune status and inflammatory response of the body through changes in their number, which may be related to tumor invasion and metastasis [24, 25]. Considering that previous studies have established prediction models on the basis of clinical data, this new discovery provides a clinical basis for further improving future models [26–28].

In terms of optimal habitat region segmentation, this study used cluster analysis to segment CT images at the voxel level during the arteriovenous phase. After careful comparison and evaluation of different clustering schemes, we found that subdividing the habitat regions into five subregions optimally maintained data integrity and internal structure. By comparing the original CT images with these finely segmented habitat regions, we observed that the Habitat1 and Habitat5 subregions were mainly concentrated around the necrotic areas of the tumor. This important discovery opens a new research perspective for exploring tumor heterogeneity.

When comparing the performance of different prediction models, we found that models based on habitat subregions had significant advantages in terms of prediction accuracy. The results of the Habitat1 and Habitat5 models in predicting the lymph node status of ICC patients were satisfactory. This advantage may stem from the superior ability of these models to capture tumor heterogeneity and the potential biological characteristics of lymph node metastasis. We combined the features of the Habitat1 and Habitat5 models to construct a stable and high-performing prediction model. By applying the model to clinical scenarios, we successfully provided lymph node status predictions for ICC patients who did not undergo lymph node dissection during surgery. Survival analysis revealed that the median survival time of patients who did not undergo lymph node dissection was significantly shorter than that of patients who did. In addition, the prognosis of the LNM + group was poor, regardless of whether lymph node dissection was performed. However, despite our initial success in applying the model to predict ICC patients without lymph node dissection and observing certain trends in survival analysis, these differences did not reach statistical significance owing to limitations in follow-up data. This suggests that we need to expand the sample size and conduct longer follow-up to fully validate the model’s predictive effectiveness and clinical application potential.

In previous studies, Ji et al. [29] extracted image features from arterial phase CT images, whereas Xu et al. [30] extracted features from T1-weighted enhanced MR images. Both studies effectively combined radiomic features with various risk factors to develop models for predicting lymph node metastasis, achieving remarkable results. However, Zhang et al. [31] constructed a fusion model of CT features by integrating radiomic features from multisequence CT images, and the performance of this fusion model was significantly better than that of radiomics models based only on single-phase CT images. On the basis of these previous studies, to construct a more robust model, we specifically selected patients with mass-forming intrahepatic cholangiocarcinoma as our study subjects. These tumors have clear boundaries, which can significantly reduce interobserver variability. Compared with traditional radiomic studies, the method of this study represented significant innovations. Through meticulous data processing and model validation, the obtained model showed a positive trend. This innovative exploration provides new ideas for radiomics research and a basis for the subsequent application of industrial models. Therefore, we expect this method to inject new vitality into radiomics research and promote the development of increased efficiency and accuracy.

In this study, a cross-validation strategy was implemented to partition the dataset scientifically, ensuring the randomness and balance of data partitioning. Although this approach comes with a high risk of overfitting, these trade-offs are necessary to pursue the high generalizability of the model. After screening, models with superior performance were identified, and their predictions were satisfactory. Nonetheless, this study has certain limitations. First, owing to the limited follow-up time, the survival data of some patients may not have reached a steady state, which may have affected the results of the survival analysis. In the future, we will continue to refine and extend follow-up. Second, although the model performs well on existing datasets, further validation is needed to assess its performance when generalized to larger and more diverse patient populations. Third, although the tumors in this study were mass-forming and were delineated and reviewed by two radiologists, manual delineation of the ROI inevitably involves some error and subjectivity. In the future, we will train an automatic segmentation model on a large sample size to identify tumor regions more accurately. Fourth, this study focused mainly on the quantitative features of radiomics and some clinical quantitative features, but there was a relative lack of attention given to some clinical qualitative features. Finally, this study focused mainly on the prediction of lymph node status, and future research can further explore the application value of this model in predicting other clinical indicators, such as distant metastasis.

## Conclusions

In summary, this study successfully constructed a prediction model based on the radiomic features of habitat subregions through deep exploration of imaging data from ICC patients, with the aim of accurately determining the lymph node status of patients. This achievement provides not only a new perspective and method for the clinical diagnosis and treatment of ICC but also an important reference and inspiration for future research.

## Supplementary Information

Supplementary Material 1.

Supplementary Material 2.

## Data Availability

The datasets used and/or analysed during the current study are available from the corresponding author on reasonable request.

## Abbreviations

ICC: Intrahepatic cholangiocarcinoma
LNM: Lymph node metastasis
CT: Computed tomography
OS: Overall survival
ROIs: Regions of interest
CH Index: Calinski–Harabasz Index
AUC: Area under the curve
DCA: Decision curve analysis
AFP: Alpha-fetoprotein
CA199: Carbohydrate antigen 199
