# Supplementary material for: To accurately predict lymph node metastasis in patients with mass-forming intrahepatic cholangiocarcinoma by using CT radiomics features of tumor habitat subregions
Source: Cancer Imaging. 2025 Feb 26;25:19. doi: 10.1186/s40644-025-00842-8 (PMC11863903; doi:10.1186/s40644-025-00842-8)
Supplement: Supplementary file 1 — Supplementary Material 1. [file 40644_2025_842_MOESM1_ESM.docx]

1. **More scan details：**

Scanning parameters for CT abdomen include: tube voltage of 120 kV, automatic tube current, rotation time of 0.4, 0.5 or 0.75 s; detector collimation of 64 x 0.625 mm, 128 x 0.625 mm or 16 x 0.625 mm, field of view of (300-500) mm x (300-500) mm, matrix of 512 x 512, layer spacing of 1mm or 1.25mm, layer thickness of 1mm or 1.25mm. After CT scanning of the abdomen, an enhanced CT scan of the abdomen was performed, starting 25-30 seconds after arterial phase contrast injection and 60-70 seconds after intravenous phase contrast injection, with intravenous injection of contrast agent (ultravist 370, Bayer Schering Pharma, Berlin, Germany) at a dose of 1.5 ml/kg and an injection rate of 3.0 to 3.5 ml/s using a high-pressure injector Ulrich CT Plus 150 (Ulrich Medical, Ulm, Germany).

Machine brand and type of CT scanning instrument: GE Healthcare (Spiral CT), GE Healthcare (Multi-slice CT), Philips Healthcare (Multi-slice CT Scanner), Siemens Healthineers (Standard CT).

1. **Additional information on habitat subregional division:**

From the already registered arterial phase and venous phase CT image sequences, the tumor region was accurately segmented according to the outlined masks to ensure that only the voxels within the tumor were included. Once the tumor regions have been segmented, the next step is to construct the grayscale matrix based on the voxels of these regions. The elements in each matrix represent the gray value of the corresponding voxel. After the construction of the gray matrix, in order to improve the subsequent clustering effect, the gray value needs to be normalized. Before clustering can begin, it needs to be determined that this clustering is performed at the cohort level to ensure that the cluster assignment is consistent across patients. Secondly, it is necessary to select the initial cluster center, which is 2 at the beginning of this study. After determining the initial cluster center, the K-means algorithm continuously updates the cluster center through an iterative process and assigns each voxel to the nearest cluster center. After each iteration, the CH Index (Calinski-Harabasz Index) was used to assess the quality of the current clustering results.

$$CH\left( K \right)=\frac{SSB/(K-1)}{SSW/(n-K)}$$

Here, SSB stands for the Sum of Squares Between Classes, SSW stands for the Sum of Squares Within Classes, n represents the total number of samples, and K represents the number of clusters. A higher CH index indicates a better clustering result.

1. **Supplementary table 1.**

| Variable | | Volume (percentage) |
| --- | --- | --- |
| Sex | male | 31(51.8) |
|  | female | 30(49.2) |
| Age | <60 | 20(32.8) |
|  | >=60 | 41(67.2) |
| HBsAg | - | 38(62.3) |
|  | + | 23(37.7) |
| AFP | <7 ng/ml | 43(70.5) |
|  | >=7 ng/ml | 18(29.5) |
| CEA | <5 ng/ml | 39(63.9) |
|  | >=5 ng/ml | 22(36.1) |
| CA199 | <27 ng/ml | 25(41.0) |
|  | >=27 ng/ml | 36(59.0) |
| Albumin | < 43.2 g/l | 48(78.7) |
|  | >= 43.2 g/l | 13(21.3) |
| GGT | < 97.9 u/l | 34(55.7) |
|  | >= 97.9 u/l | 27(44.3) |
| ALP | < 208.2 u/l | 47(77.0) |
|  | >=208.2 u/l | 14(23.0) |
| INR | <1.025 | 37(60.7) |
|  | >=1.025 | 24(39.3) |
| APTT | <29.1 s | 39(63.9) |
|  | >=29.1 s | 22(36.1) |
| WBC | <6.7 10*9/l | 29(47.5) |
|  | >=6.7 10*9/l | 32(52.5) |
| NC | <4.2 10*9/l | 25(41.0) |
|  | >=4.2 10*9/l | 36(59.0) |
| LC | <1.8 10*9/l | 47(77.0) |
|  | >=1.8 10*9/l | 14(23.0) |
| PLT | <280.5 10*9/l | 49(80.3) |
|  | >=280.5 10*9/l | 12(19.7) |
| Creatinine | <51.5 umol/l | 29(47.5) |
|  | >=51.5 umol/l | 32(52.5) |

HBsAg, Hepatitis B Surface Antigen; AFP, Alpha-Fetoprotein; CEA, Carcinoembryonic Antigen; CA199, Carbohydrate Antigen 199; GGT, Gamma-Glutamyl Transferase; ALP, Alkaline Phosphatase; INR, International Normalized Ratio; APTT, Activated Partial Thromboplastin Time; WBC, White blood cell count; NC, Neutrophil count; LC, Lymphocyte count; PLT, Platelet count.

1. **Supplementary table 2. Results of the delong test for model comparisons**

|  | Significance (P value) | Standard error difference | 95% CI Lower Limit | 95% CI Upper Limit |
| --- | --- | --- | --- | --- |
| INTRA - H5 | .011 | .271 | -.226 | -.029 |
| INTRA - H4 | .274 | .288 | -.165 | .047 |
| INTRA - H3 | .360 | .311 | -.075 | .207 |
| INTRA - H2 | .658 | .303 | -.098 | .155 |
| INTRA - H1 | .598 | .298 | -.139 | .080 |
| INTRA - H5+1 | .004 | .267 | -.228 | -.044 |

CI, confidence interval.
